# Relative Echogenicity of Tendons and Ligaments of the Palmar Metacarpal Region in Foals from Birth to 4 Months of Age: A Longitudinal Study

**DOI:** 10.1371/journal.pone.0159953

**Published:** 2016-07-21

**Authors:** Giuseppe Spinella, Domenico Britti, Giovanni Loprete, Vincenzo Musella, Noemi Romagnoli, Jose M. Vilar, Simona Valentini

**Affiliations:** 1 Department of Veterinary Medical Sciences, University of Bologna, Ozzano dell'Emilia (BO), Italy; 2 Department of Health Sciences, University Magna Graecia of Catanzaro, Germaneto (CZ), Italy; 3 Department of Animal Pathology, Faculty of Veterinary Medicine, University of Las Palmas de Gran Canaria, Trasmontaña S/N, Arucas, Las Palmas, Spain; University of Bari Aldo Moro, ITALY

## Abstract

The objective of this study was to evaluate relative echogenicity of superficial and deep digital flexor tendons, the accessory ligament of the deep digital flexor tendon and interosseous muscle of the metacarpal region in foals ages 1 week to 4 months; and assess the association between echogenicity and sex or side/laterality. Seven Standardbred trotter foals were examined. Right and left metacarpal regions (palmar surface) were ultrasonographically investigated, and four regions of interest were assessed. A significant increase in echogenicity was seen in superficial and deep digital flexor tendons, accessory ligament of deep digital flexor tendon, and interosseous muscle during growth from 1 week to 4 months of age. Echogenicity of examined tendons and ligaments was not influenced by gender nor laterality. Reference values for tendon and ligament echogenicity could function as a tool to discriminate between physiological and abnormal conditions such as congenital contractural conditions.

## Introduction

Interest in ultrasound qualitative and quantitative methods for tendon and ligament lesion evaluation has recently increased in veterinary medicine [1–5]. Current quantitative methods to evaluate structural integrity and size of tendons are mean echogenicity (ME), relative echogenicity (RE) and cross-sectional area (CSA). Quantitative methods allow objective evaluation of the structural integrity of tendons through appropriate measurements, using specific software [2,4,6–8]. These methods numerically express ultrasonographic quantitative features, providing quicker evaluation of normal and pathological conditions. In adult horses, quantitative ultrasonographic evaluation of flexor tendons and ligaments in the metacarpal region has been widely described [2,4,9]. A preliminary report has described ME in neonatal foals [10], but no publications report tendon and ligament RE in foals during growth. RE is the ratio of ME of a region of interest (ROI) to ME of a structure with reproducible echogenicity. RE was previously calculated by Agut et al. by dividing tendon or ligament ME by the mean ME value for an area of the same size and depth using free phantom imaging [2]. A simpler method was suggested by Padilla et al., who calculated RE = (mean pixel brightness [PB] of ROI) / (PB of bone) × 100 [11].

For objective evaluation, instrument variables, such as amplifier gain level, transducer tilt, and transducer displacement, need to be standardized [12]. The knowledge of reference values for tendon and ligament echogenicity and their changes during growth could function, in the authors’ opinion, as a tool to discriminate between physiological and abnormal conditions, also providing prognostic information in subjects affected by muscular or tendon contractures during growth.

The objectives of this preliminary study were to evaluate RE of the superficial and deep digital flexor tendons (SDFT and DDFT), accessory ligament of deep digital flexor tendon (ALDDFT), and suspensory ligament or interosseous muscle (IM) [13] of the metacarpal region in foals from 1 week to 4 months of age, and to determine the effects of sex and laterality (left and right limb) on this quantitative ultrasonographic method. For RE evaluation, our aim was to add information about ultrasound quantitative methods in foals, and not to compare the validity of this method with ME and/or CSA.

## Materials and Methods

### Animals

The right and left metacarpal regions (palmar surfaces) of seven orthopedically sound, client-owned foals were examined from birth (within 1 week of age) to 4 months of age, at regular intervals of 30 days. Foals were maintained on box rest at night and kept at pasture during the day. Before ultrasound examination, a physical examination was performed on each foal, including inspection, palpation, and dynamic and static flexion of the forelimbs (left and right), in order to exclude any congenital or acquired condition affecting the musculoskeletal system. All procedures on the animals were carried out with the approval of the Ethical Committee of the University of Bologna (Project ID 9-79-2014), in accordance with DL 116/92, approved by the Ministry of Health. Oral informed consent was given by the owners.

### Ultrasound procedure

The ultrasound procedure was performed on standing, non-sedated foals with a portable, real-time ultrasound scanner (MyLab One/Touch Ultrasound System, Esaote, Genoa, Italy) with a multifrequency linear probe (10–13 MHz). To standardize the exam, each procedure was performed at 10 MHz frequency, with 50% gain resolution and a 50-mm deep examination probe with focus set at 1.0, 2.0, or 2.5 cm (for SDFT, ALDDFT/DDFT, and IM, respectively). The metacarpal region was washed with water and ethyl alcohol; no shaving was performed. To allow consistent identification of flexor tendons and ligaments of the metacarpal region, four areas were identified (1A, 1B, 2A, 2B), according to Rantanen classification [14] adapted to the foal, starting distally to the midpoint of the accessory carpal bone. Transverse scans of the SDFT, DDFT, ALDDFT, and IM were obtained. The ROI for evaluation of ME and RE was identified with the perimeter of each examined tendon or ligament in transverse scan.

Ultrasound images were digitized, and ME of the ROI (defined as mean pixel brightness or mean grey level of the tendon/ligament area) was calculated using an image processing software (ImageJ, National Institutes of Health, Bethesda, Maryland, USA) with a scale of 256 grey levels (0 = black; 255 = white). Grey distribution appears as a histogram, and mean value is automatically calculated by the software. On each forelimb of each horse, ME of flexor tendons and ligaments was calculated. A blind evaluation was performed by two authors for the presence of abnormalities. All images were considered as normal or not pathologic, and three measurements at each level for each tendon and ligament were made. For reproducibility analysis, images from each ROI were delineated and measured by the two observers. Additionally, ME of the metacarpal bone at the same level of the studied ROIs was calculated; for a more accurate evaluation of this last parameter, mean value of 10 measurements was used. RE was then calculated as follows [11]:

RE=(tendon or ligament ME )/(ME bone)  × 100

### Statistical analysis

Intra- and inter-observer reproducibility of agreement was estimated by Bland-Altman and Spearman methods. The Shapiro-Wilk test was applied to verify normal distribution of data and residuals; subsequently, RE differences between different examined structures, along with different ROIs for the same structure at different ages, were analyzed using analysis of variance with the Bonferroni-Dunn test for post hoc comparisons. The influence of sex (male and female) and laterality (left and right forelimb) on echogenicity was also analyzed with the Student *t* test. Values of *P* ≤ 0.05 were accepted as significant for all tests.

## Results

**Fig 1:**
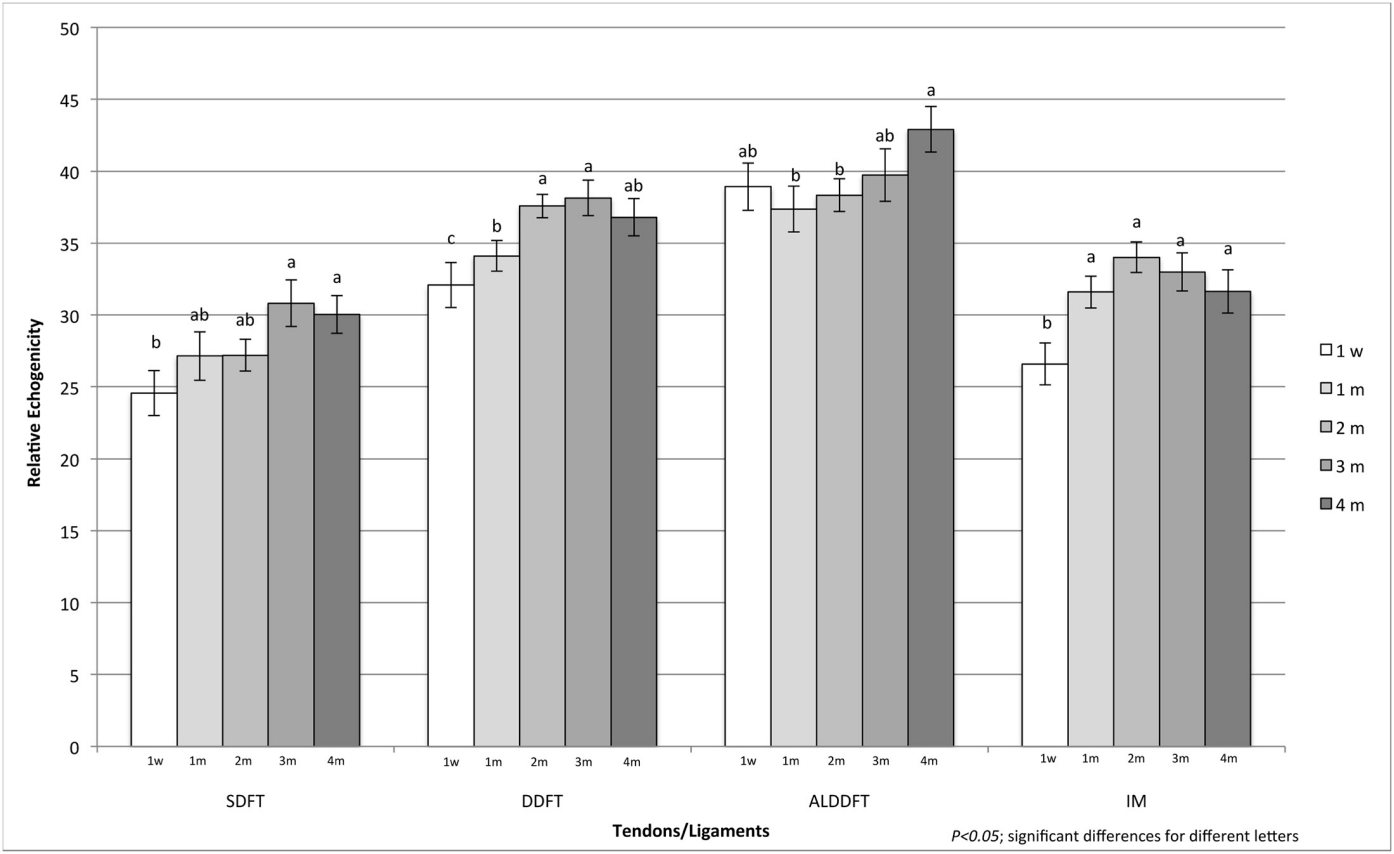
“Mean” Relative echogenicity and standard errors of examined foal tendons analyzed in their entirety along four regions of interest in relation to age. Abbreviations: DDFT = deep digital flexor tendon; SDFT = superficial digital flexor tendon; IM = interosseous muscle; ALDDFT = accessory ligament of deep digital flexor tendon; w = week; m = month. Differing letters indicate significant differences (*P*<0.05).

**Fig 2:**
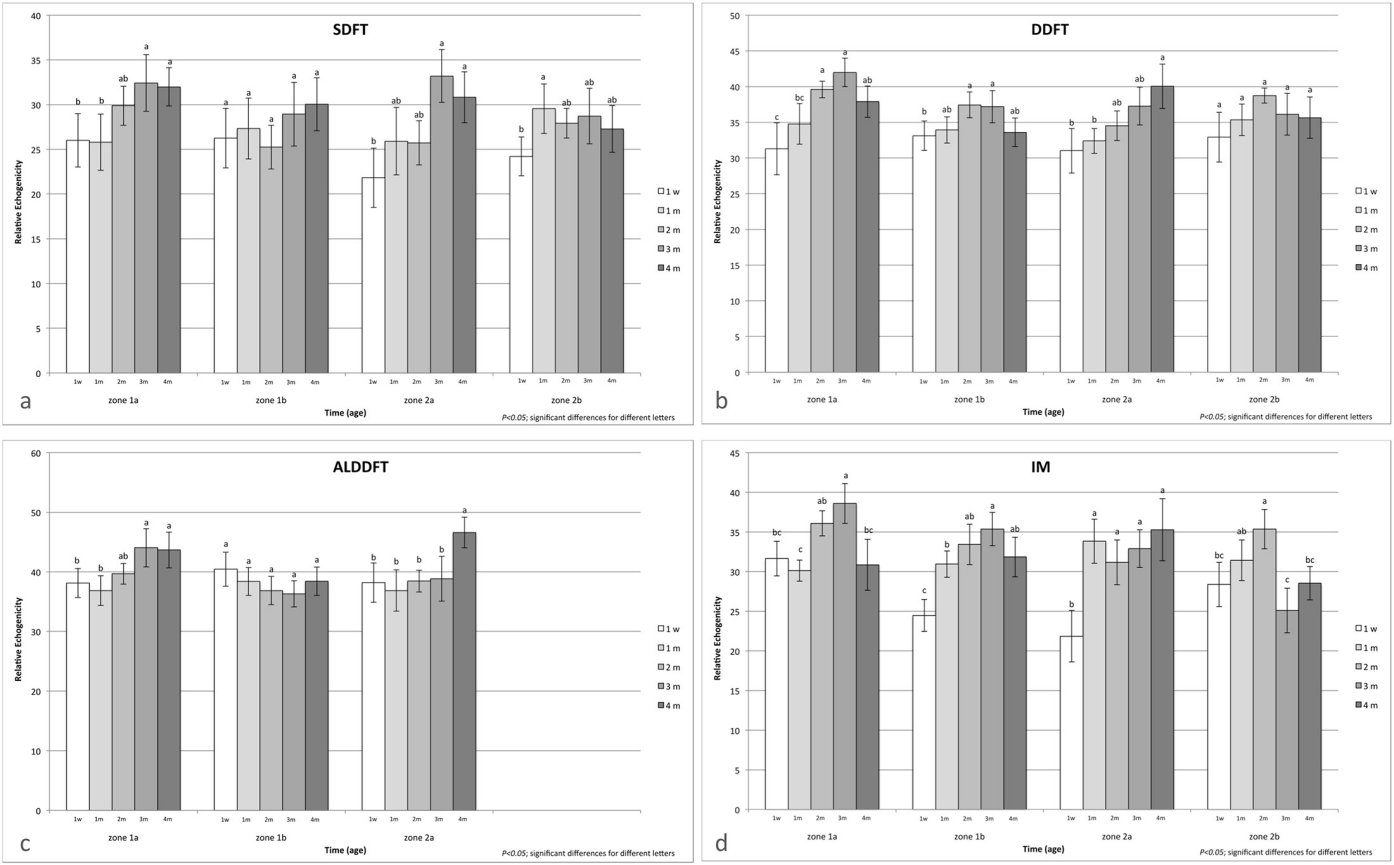
“Mean” Relative echogenicity and standard errors of superficial digital flexor tendon (SDFT) (a), deep digital flexor tendon (DDFT) (b), accessory ligament of deep digital flexor tendon (ALDDFT), and interosseous muscle (IM) in each region of interest in relation to age of foals. Differing letters indicate significant differences (*P*<0.05). Abbreviations: w = week; m = month.

Seven Standardbred trotter foals (four males and three females) were included in the study. Reproducibility and agreement tests between operators gave a result of 97% and 0.8 correlation (very good). The mean value of the sum of RE of each ROI was obtained for all examined structures (SDDF, DDFT, ALDDFT, IM) and showed a significant increase in echogenicity during growth from 1 week to 4 months of age (Fig 1). The DDFT and ALDDFT were the most echogenic structures, with higher RE values. Regarding sex and laterality, there was no significant difference in RE between males and females nor between right and left forelimb. Otherwise, increases of RE for each structure (SDDF, DDFT, ALDDFT, and IM) were observed from 1 week to 4 months of age in different ROIs. The RE increase was statistically significant for the SDFT in ROIs 1A, 2A, and 2B; for the DDFT in ROIs 1A, 1B, and 2A; for the ALDDFT in ROIs 1A and2A; and for the IM in all ROIs considered (Fig 2).

## Discussion

The importance of ultrasonographic measurement of ME and RE in horses and dogs has been well established [2,8,15], but to the authors’ knowledge, this is the first publication reporting information regarding RE tendon measurements in foals during growth.

The present study on echogenicity changes was performed in order to create a preliminary semiological database that could be used not only to identify and quantify a pathological lesion, but also to investigate hypothetical prognostic information for tendon and ligament contracture development in the most important region of the locomotor system in horses. Effectively, flexural deformities occurring in foals often result in shortening of the musculotendinous unit of the DDFT [16]. Congenital deformities themselves are often a consequence of improper nutritional management of the mare during gestation or malpositioning of the fetus in utero [17]. Acquired deformities generally develop in foals from 2 to 6 months of age; although the etiology of acquired deformities is unknown, speculated causes include genetic predisposition, improper nutrition, and excessive exercise [16]. Moreover, Van Heel et al. reported that the tendency of foals to develop an acquired flexural deformity is related to particular physical characteristics such as long legs and short necks [18].

In this study, RE was investigated using the method proposed by Padilla et al. for brain in children [11], where RE = (ME)/(ME bone) × 100. Agut et al. and, previously, Micklethwaite et al. [2,19] compared the value of ME of tendons and ligaments with the echogenicity values of three different phantoms. In the opinion of the authors, even if this last method could produce more objective results, it is not easily applicable in clinical equine practice. Padilla et al.’s method appeared to be more feasible, easier for clinicians to apply, and less expensive because a structure with known echogenicity (bone) was present in our recorded ultrasound image and, consequently, phantoms’ building was not required.

Our study has emphasized a scale of echogenicity of the teno-desmic structures with lower echogenicity of the SDFT and an interesting and unexpected, statistically significant variation for echogenicity in IM. In most adult horses, IM contains very little muscular tissue, although the amount of muscle tissue may vary between the forelimbs and the hind limbs of the same horse or among breeds [14]. On the other hand, Wilson et al. reported no statistically significant difference in the percentage of muscle content of the IM with regard to the specific age of the horse, but a substantial amount of muscle in the proximal segments of the IM [20]. This difference may be because during weight-bearing, the IM is under greater tension and has more of its collagen fiber bundles available to the ultrasonographic beam than other tendons and ligaments [2]. In our findings, even ME and RE values for IM were lower compared to DDFT and ALDDFT, but these differences were not statistically significant. However, it is our opinion that the lower IM echogenicity observed in neonatal foals and the increase seen from birth to 4 months of age are clear indications of a decreasing amount of muscle fibers, starting from the distal part of the IM body close to the origin of the branches. Nevertheless, as previously reported [20], it has been demonstrated that in adult horses, the IM has an echogenicity similar to that of a ligament, limiting its similarity to muscle.

In this study, the SDFT was the only tendon with a significantly lower echogenicity compared with other examined structures. This finding has been widely justified in adult horses. Dowling and Dart reported that this feature was related to a non-collagenous matrix, which allowed an adaptive response to exercise in tendons in young horses that decreased when horses reach maturity [21]. Three types (I, II, III) of tenocytes have been identified within the equine tendon; type II cells predominate in fetal tendons and are metabolically more active and responsive for matrix maintenance [21]. With increasing age, the cell population radically changes, and type I cells predominate, with a lower age-associated metabolic activity of this collagen type. Moreover, low echogenicity of SDFT could be related to the cellularity and fiber undulation observed in very young foals [22]. Thus, in foals, cellularity was high with a total absence of focal chondroid metaplasia (fibrocartilaginous matrix) [22].

Lastly, no significant difference between the echogenicity of the tendons and ligaments of left and right forelimbs and between males and females during growth were detected. These findings were in agreement with previous studies performed in adult horses [2,15].

A limit of this study was the small number of patients enrolled; this number would obviously need to be increased to create a more reliable database. Moreover, the tendon artefact, known as anisotropy, could have limited the accuracy of our results. This artefact could be avoided only with perfect control of the transducer in a perpendicular position (90°), avoiding tilt and displacement, as described in an ex vivo study by Van Schie et al. [12]. In the authors’ opinion, this condition was unlikely achievable in practice. However, further histologic and echographic evaluation of the tendons could better clarify this issue.

As reported for muscle contracture, it is presumable that structures affected by contracture will have higher echogenicity for fibrous tissue replacement, and that this echogenicity will be more easily detectable with quantitative ultrasonographic parameters.

In conclusion, it is authors’ opinion that ultrasound investigation and RE evaluation of metacarpal tendons and ligaments in Standardbred foals could be crucial tools both for diagnosis of pathological conditions and in early diagnosis of flexural and angular limb deformities. The measurements obtained in Standardbred foals in this study are highly specific for this breed, and other equine breeds should be further investigated.

## References

[1] Spinella G, Valentini S, Tamburro R, Capitani O. Examen échographique des structures musculaires du chien. Point Vet. 2007; 38:31–34.

[2] Agut A, Martınez ML, Sanchez-Valverde MA, Soler M, Rodríguez MJ. Ultrasonographic characteristics (cross-sectional area and relative echogenicity) of the digital flexor tendons and ligaments of the metacarpal region in Purebred Spanish horses. Vet J. 2009; 180:377–383. 10.1016/j.tvjl.2008.01.012 18400531

[3] Dakin SG, Jespers K, Warner S, O'Hara LK, Dudhia J, Goodship AE, et al The relationship between in vivo limb and in vitro tendon mechanics after injury: a potential novel clinical tool for monitoring tendon repair. Equine Vet J. 2011; 43:418–423. 10.1111/j.2042-3306.2010.00303.x 21496076

[4] Vilar JM, Santana A, Espinosa J, Spinella G. Cross-sectional area of the tendons of the tarsal region in Standardbred trotter horses. Equine Vet J. 2011; 43:235–239. 10.1111/j.2042-3306.2010.00141.x 21592221

[5] Vergari C, Pourcelot P, Ravary-Plumioën B, Dupays AG, Denoix JM, Mitton D, et al First application of axial speed of sound to follow up injured equine tendons. Ultrasound Med Biol. 2012; 38:162–167. 10.1016/j.ultrasmedbio.2011.10.008 22104528

[6] Van Schie HTM, Bakker EM, Jonker AM, van Weeren PR. Ultrasonographic tissue characterization of equine superficial digital flexor tendons by means of grey level statistics. Am J Vet Res. 2000; 61:210–219. 10685695 10.2460/ajvr.2000.61.210

[7] Van Schie HTM, Bakker EM, Jonker M, van Weeren PR. Computerized ultrasonographic tissue characterization equine superficial digital flexor tendons by means of stability quantification of echo patterns in contiguous transverse ultrasonographic images. Am J Vet Res. 2003; 64:366–375. 12661879 10.2460/ajvr.2003.64.366

[8] Spinella G, Loprete G, Musella V, Britti D, Vilar J. Cross-sectional area and mean echogenicity of shoulder and elbow tendons in adult German Shepherd dogs. Vet Comp Orthopaed. 2013; 26:366–371. 10.3415/VCOT-12-11-0144 23800825

[9] Gillis C, Pool RR, Meagher DM, Stover SM, Reiser K, Willits N. Effect of maturation and ageing on the histomorphometric and biochemical characteristics of equine superficial digital flexor tendon. Am J Vet Res. 1997; 58:425–430. 9099392

[10] Spinella G, Loprete G, Castagnetti C, Musella V, Antonelli C, Vilar JM, et al Evaluation of mean echogenicity of tendons and ligaments of the metacarpal region in neonatal foals: a preliminary study. Res Vet Sci. 2015; 101:11–14. 10.1016/j.rvsc.2015.05.011 26267082

[11] Padilla NF, Enriquez G, Jansson T, Gratacos E, Hernandez-Andrade E. Quantitative tissue echnogenicity of the neonatal brain assessed by ultrasound imaging. Ultrasound Med Biol. 2009; 35:1421–1426 10.1016/j.ultrasmedbio.2009.04.014 19632762

[12] Van Schie JTM, Bakker EM, Van Weeren PR. Ultrasonographic evaluation of the equine tendons: a quantitative in vitro study of the effects of amplifier gain level, transducer-tilt, and transducer-displacement. Vet Radiol Ultrasound. 1999; 40:151–160. 10225527 10.1111/j.1740-8261.1999.tb01901.x

[13] Dyce KM, Sack WO, Wensing CJG. The forelimb of the horse Textbook of Veterinary Anatomy, 4th Edition Saunders Elsevier, St. Louis Missour; 2010 pp. 604–609.

[14] Sande RD, Tucker RL, Johnson GR. Diagnostic ultrasound: applications in the equine limb In: Rantenan NW, McKinnon AO, editors. Equine diagnostic ultrasonography. Baltimore: Wiley; 1998 pp. 103–123.

[15] Gillis C, Meagher DM, Cloninger A, Locatelli L, Willits N. Ultrasonographic cross-sectional area and mean echogenicity of the superficial and deep digital flexor tendons in 50 trained Thoroughbred racehorses. Am J Vet Res. 1995; 56:1265–1269. 8928940

[16] O’Grady SE. Flexural deformities of the distal interphalangeal joint (clubfeet). Equine Vet Educ. 2012; 24:260–268.

[17] Trumble NT. Orthopedic disorders in neonatal foals. Vet Clin North Am Equine Pract. 2005; 21:357–385. 16051054 10.1016/j.cveq.2005.04.008

[18] Van Heel MC, Kroekenstoel AM, Van Dierendonck MC, van Weeren PR, Back W. Uneven feet in a foal may develop as a consequence of a lateral grazing behavior induced by conformational traits. Equine Vet J. 2006; 38:373–378. 10.2746/042516406x159070 17228580

[19] Micklethwaite L, Wood AK, Sehgal CM, Polansky M, Dowling BA, Dart AJ, et al Use of quantitative analysis of sonographic brightness for detection of early healing of tendon injury in horses. Am J Vet Res. 2001; 62:1320–1327. 11497458 10.2460/ajvr.2001.62.1320

[20] Wilson DA, Baker GJ, Pijanowski GJ, Boero MJ, Badertscher RR 2nd. Composition and morphologic features of the interosseous muscle in Standardbreds and Thoroughbreds. Am J Vet Res. 1991; 52:133–139. 2021241

[21] Dowling BA, Dart AJ. Mechanical and functional properties of the equine superficial digital flexor tendon. Vet J. 2005; 170:184–192. 16129339 10.1016/j.tvjl.2004.03.021

[22] Crevier-Denoix N, Collobert C, Sanaa M, Bernard N, Joly C, Pourcelot P, et al Mechanical correlations derived from segmental histologic study of the equine superficial digital flexor tendon, from foal to adult. Am J Vet Res. 1998; 59:969–977. 9706200

